# Self-determination theory interventions versus usual care in people with diabetes: a protocol for a systematic review with meta-analysis and trial sequential analysis

**DOI:** 10.1186/s13643-020-01566-5

**Published:** 2021-01-07

**Authors:** Anne Sophie Mathiesen, Mette Juel Rothmann, Vibeke Zoffmann, Janus Christian Jakobsen, Christian Gluud, Jane Lindschou, Mette Due-Christensen, Bodil Rasmussen, Emilie Marqvorsen, Thordis Thomsen

**Affiliations:** 1 Department of Endocrinology, Center for Cancer and Organ Diseases, Copenhagen University Hospital, Rigshospitalet, Blegdamsvej 9, 2100 Copenhagen, Denmark; 2 Steno Diabetes Center Odense, Odense University Hospital, Odense, Denmark; 3 Department of Endocrinology, Odense University Hospital, Odense, Denmark; 4 Department of Clinical Research, University of Southern Denmark, Odense, Denmark; 5 School of Nursing and Midwifery, Faculty of Health, Deakin University, Melbourne, Australia; 6 The Research Unit Women’s and Children’s Health, The Julie Marie Center, Copenhagen University Hospital, Rigshospitalet, Copenhagen, Denmark; 7 Sector of Health Services Research, Department of Public Health, University of Copenhagen, Copenhagen, Denmark; 8 Copenhagen Trial Unit, Centre for Clinical Intervention Research, Rigshospitalet, Copenhagen, Denmark; 9 Department of Regional Health Research, The Faculty of Health Sciences, University of Southern Denmark, Odense, Denmark; 10 Faculty of Nursing, Midwifery and Palliative Care, King’s College London, London, UK; 11 Steno Diabetes Center Copenhagen, The Capital Region of Denmark, Copenhagen, Denmark; 12 Department of Anaesthesiology, Herlev University Hospital, Herlev, Denmark; 13 Department of Clinical Medicine, University of Copenhagen, Copenhagen, Denmark

**Keywords:** Type 1 diabetes, Type 2 diabetes, Self-determination theory, Guided self-determination method, Quality of life, Diabetes distress, Depressive symptoms, Glycated haemoglobin, Health education tools, Psychosocial support

## Abstract

**Background:**

Existing self-management and behavioural interventions for diabetes vary widely in their content, and their sustained long-term effectiveness is uncertain. Autonomy supporting interventions may be a prerequisite to achieve ‘real life’ patient engagement and more long-term improvement through shared decision-making and collaborative goal setting. Autonomy supportive interventions aim to promote that the person with diabetes’ motivation is autonomous meaning that the person strives for goals they themselves truly believe in and value. This is the goal of self-determination theory and guided self-determination interventions. Self-determination theory has been reviewed but without assessing both benefits and harms and accounting for the risk of random errors using trial sequential analysis. The guided self-determination has not yet been systematically reviewed. The aim of this protocol is to investigate the benefits and harms of self-determination theory-based interventions versus usual care in adults with diabetes.

**Methods/design:**

We will conduct the systematic review following The Cochrane Collaboration guidelines. This protocol is reported according to the PRISMA checklist. A comprehensive search will be undertaken in the CENTRAL, MEDLINE, EMBASE, LILACS, PsycINFO, SCI-EXPANDED, CINAHL, SSCI, CPCI-S and CPCI-SSH to identify relevant trials. We will include randomised clinical trials assessing interventions theoretically based on guided self-determination or self-determination theory provided face-to-face or digitally by any healthcare professional in any setting. The primary outcomes will be quality of life, mortality, and serious adverse events. The secondary will be diabetes distress, depressive symptoms and adverse events not considered serious. Exploratory outcomes will be glycated haemoglobin and motivation. Outcomes will be assessed at the end of the intervention and at maximum follow-up. The analyses will be performed using Stata version 16 and trial sequential analysis. Two authors will independently screen, extract data from and perform risk of bias assessment of included studies using the Cochrane risk of bias tool. Certainty of the evidence will be assessed by GRADE.

**Discussion:**

Self-determination theory interventions aim to promote a more autonomous patient engagement and are commonly used. It is therefore needed to evaluate the benefit and harms according to existing trials.

**Systematic review registration:**

PROSPERO CRD42020181144

**Supplementary Information:**

The online version contains supplementary material available at 10.1186/s13643-020-01566-5.

## Background

Diabetes affects 425 million people worldwide, and of these, type 2 diabetes accounts for 90% [1]. The prevalence and incidence of both type 1 and type 2 diabetes are rapidly increasing [1]. Likewise, the ageing population contributes to a substantial rise in the number of people with diabetes.

Type 1 diabetes is caused by an autoimmune reaction where the body’s immune system attacks the insulin-producing beta cells in the islets of the pancreas gland [1]. Consequently, the body produces little to no insulin [1]. Thus, people with type 1 diabetes depend on multiple daily insulin injections and on managing multiple self-care tasks to maintain a glucose level close to the normal range. The disease can develop at any age and around half of people with type 1 diabetes are diagnosed in adulthood [2].

Type 2 diabetes is caused by a genetic disposition in combination with a sedentary lifestyle and overweight [3]. These risk factors lead to insulin resistance, which initially prompts an increase in insulin production but causes decreased insulin secretion over time. More than 80% are overweight at the time of diagnosis [4]. The risk of developing type 2 diabetes increases from 50 to 75% when one or both parents, respectively, have type 2 diabetes [5]. With age being the single largest risk factor for developing type 2 diabetes, the number of people living with type 2 diabetes and various combinations of comorbidities is also increasing.

Complications to diabetes include macrovascular complications such as ischaemic stroke or coronary heart disease [6]. Microvascular complications comprise retinopathy, neuropathy, and nephropathy [7]. Whilst tight glycaemic control is associated with reduced microvascular complications [6, 7], this association is less clear in relation to macrovascular complications [8]. Due to the early onset of type 1 diabetes, complications of type 1 diabetes are more susceptible to develop [9]. In people with type 2 diabetes, macrovascular complications are associated with age, male sex, obesity, dyslipidaemia, and smoking [10]. Up to one-third of people with type 2 diabetes have developed one or more complications of type 2 diabetes at the time of diagnosis [10]. A longer pre-detection period for unrecognised type 2 diabetes in people with low educational status infers a prolonged time for the complications of diabetes to develop [11].

Unhealthy lifestyle behaviours and body mass index may explain up to 45% of the social inequality in type 2 diabetes [4]. Within type 1 diabetes, no social gradient exists [9], but still some interventions may potentially broaden the gap between social groups [12–15]. In this systematic review, we plan to investigate a potential differential impact of included interventions because social inequality is an issue in people with type 2 diabetes. As the interventions under investigation require a level of literacy and language skills they may potentially further increase inequity [14].

The human and economic drain from diabetes is excessive; not only because of direct costs but also due to indirect costs like managing complications of diabetes, sick days and early retirement. From a socioeconomic viewpoint, there appears to be convincing incentives to invest in people with diabetes and comorbidities, as the expenses for each individual disease may accumulate, resulting in total costs that exceed the expenses for each individual disease [16]. Due to this effect, the return of investment is often underestimated when intervening in these patients [16].

### Description of the interventions

Diabetes self-management defined as leading a healthy lifestyle, measuring blood glucose, taking medicine and receiving support from healthcare professionals and own social network is considered crucial to reduce development of complications of diabetes and increase quality of life [17].

The diabetes management plan for people with diabetes should consider the person’s age, cognitive abilities, literacy, social and financial situation, cultural factors, diabetes complications and comorbidities, health priorities and preferences of care [18]. Autonomy supporting interventions may lead to satisfactory diabetes self-management because lifestyle changes are easier to accomplish and maintain if the person’s motivation is autonomous meaning that they strive for goals they themselves truly believe in and value [19]. Thus, autonomy interventions may be a prerequisite to achieve ‘real life’ engagement and more long-term improvement of the person with diabetes through shared decision-making and collaborative goal setting. Within autonomy interventions, intrinsic motivation is a key concept as it is connected to success to reach and sustain treatment targets [20, 21].

Existing diabetes self-management interventions and interventions focusing on behaviour change vary widely in their content, and their sustained long-term effectiveness is uncertain [22, 23]. Reviews suggest that interventions that are grounded in behavioural change theory are more effective than those that are not [21, 24]. Educational interventions, psychological interventions and health educational tools are based on different theoretical grounding, training, clinical skills, and are delivered by different specialists in diverse settings. Educational interventions use didactic and enhanced learning methods to improve self-management of diabetes by reducing identifiable gaps in knowledge [25]. Psychological therapies use the therapeutic alliance between patient and therapist, in which the patient’s problems are understood in terms of emotions, cognitions, and behaviours [26], yet, psychological interventions have not proven effective on glycated haemoglobin (HbA1c) in people with type 1 [27] or type 2 diabetes [22]. Health educational tools that aim at translating person-centred care into practice and finding ways to enhance intrinsic motivation may lead to greater long-term behaviour change than tools solely relying on external motivation [28]. This is the goal of the guided self-determination method [29–32] and self-determination theory [33].

We aim to assess the effects of the autonomy-supportive inventions: guided self-determination intervention by Zoffmann [29–31] and self-determination theory by Deci and Ryan [33]. Guided self-determination is an empowerment-based method recognised as a life-skills approach clinically applicable in patient-provider relationships. The guided self-determination method was empirically developed on the basis of grounded theory [29–32] and formal theories including self-determination theory and life skills theory. The self-determination theory is based on comprehensive empirical research [33]. For transparency, the guided self-determination method and self-determination theory are described according to the model of analyses based on the criteria proposed by Graham et al. [34–36] shown in Supplementary Material Table 1.

### How the interventions might work

In the guided self-determination approach, the person with diabetes has a primary role preparing for consultations at home, filling in reflection sheets. This means that the person needs to clarify and prioritise what is important to change, thus becoming able to express their thoughts in communication with the healthcare professionals. Guided self-determination intervention is likely to improve clinical outcomes through the following pathways [32, 37]: increased perceived autonomy support from the healthcare professionals, a higher frequency of self-monitored blood glucose, increased perceived competence in managing diabetes, decreased diabetes-related distress and ultimately improved glycaemic control [29–32].

According to the self-determination theory, when the three basic psychological needs: competence, autonomy and relatedness are satisfied; this leads to enhanced autonomous motivation and mental health [33, 38, 39]. Self-determination theory proposes a continuum for the internalisation of motivation, whereby people become more autonomous (or self-determined) to engage in behaviours over time. The pathways of mechanisms are built on a theoretical model [33], which argues, first, that social-contextual events (e.g. feedback, communications, rewards) that conduce towards feelings of competence during action can enhance intrinsic motivation for that action. Accordingly, optimal challenges, tailored feedback, and lack of demeaning evaluations are hypothesised to facilitate intrinsic motivation and thereby promote autonomy [33].

### Why is it important to do this systematic review

We conducted preliminary literature searches in PubMed and the Cochrane Database of Systematic Reviews using the search terms: diabetes, theory-based interventions, self-determination theory, guided self-determination and person-centred in different combinations. From these searches, we identified three reviews including studies that provided self-determination theory for behaviour change in the health domain [20, 21, 40]. None of the reviews were systematic reviews. The three reviews investigating self-determination theory [20, 21, 40], included trials investigating the effect of the self-determination theory-based intervention assessing at least one self-determination theory variable. All three reviews [20, 21, 40] included trials from different populations, primarily with healthy people and multiple experimental designs. Nevertheless, whether an improvement can be attributed to the intervention, it can only be established in randomised clinical trials. An overview of the characteristics of the three reviews is shown in Table 1. None of the reviews had a registered or published protocol, none were based on unrestricted searches and bias risk was only assessed in two reviews, using domains adopted from the Cochrane Handbook for conducting and reporting systematic reviews and meta-analyses [52, 53]. None of the reviews controlled the risks of random errors using trial sequential analysis, the outcomes reported were limited to specific self-determination theory constructs, and none of the reviews assessed adverse effects. In the review of Ntoumanis et al. [20], the authors concluded that changes in autonomous motivation and perceptions of need support were associated with small positive changes in health behaviours at the end of the intervention, but small to medium changes at follow-up, which may indicate the potential of a sustained behaviour change [20].

**Table 1:** Self-determination theory reviews

	Ng et al. [40]	Gillison et al. [21]	Ntoumanis et al. [20]
Designs included	184 independent datasets, primarily non-experimental design	74 studies including a control group (59 of randomised clinical trials (RCTs) or cluster RCTs)	73 independent datasets, 58 RCTs (20 of these were cluster RCTs)
Number of trials including people with diabetes	Seven trials [32, 41–46]	One trial [47]	Six trials [45, 47–51]
Registered in PROSPERO	No	No	No
Protocol published	No	No	No
Restricted searches	PsycINFO, PsycARTICLES and PubMed, citation searches (ISI web of knowledge)	Web of Science, PsychINFO, PubMed, Cochrane Database, DARE, Biomed Central, Sociological abstracts, ProQuest	PsycINFO, PsycARTICLES and PubMed/Medline
Assessment of bias risk	Not performed	A modified version of the Cochrane risk of bias tool (random group allocation, treatment allocation concealment, groups similar at baseline, blinded outcome assessor, intention-to-treat analyses, risk of bias)	A modified version of the Cochrane risk of bias tool (random group allocation, group allocation concealment, blinded outcome assessor, handling of missing data, selective reporting, other bias)
Assessment of random errors, using trial sequential analysis	No	No	No
Outcomes	Specific self-determination theory constructs	Specific self-determination theory constructs	Specific self-determination theory constructs
Assessment of adverse effects	No	No	No

Regarding guided-self-determination, we found no systematic reviews but we identified four randomised clinical trials providing guided self-determination for people with diabetes [32, 50, 54, 55]. Of these, one randomised clinical trial investigating the effect of guided self-determination in young adults with type 1 diabetes identified a larger effect on HbA1c and diabetes distress at follow-up compared to immediately after the intervention in women, but not in men [54]. Due to the limitations of the existing reviews outlined in Table 1 and the fact that guided self-determination intervention method had not yet been systematically reviewed, we find it justified to conduct a systematic review including trial sequential analysis and GRADE for assessing the potential of a long-term effect, specifically targeting people with diabetes.

### Objective

The objective is to investigate the benefits and harms of guided self-determination and self-determination theory interventions versus usual care in people with diabetes.

## Methods

This protocol has been registered in the PROSPERO database ID nr. CRD42020181144 on 5 July 2020, and is reported according to the Preferred Reporting Items for Systematic reviews and Meta-analysis Protocols (PRISMA-P) 2015 statement [56] (Checklist as Additional file 1).

### Criteria for considering studies for this review

#### Types of studies

We will include randomised clinical trials and cluster randomised trials irrespective of publication status, reported outcomes, publication date, publication type, and language conducted in any setting for assessment of benefits and harms. We will not include quasi-randomised studies or observational studies [52].

#### Types of participants

People with a diagnosis of type 1 diabetes or type 2 diabetes as defined by trialists. The participants should be described as adolescents or adults by trialists. Trials including participants described as children will be excluded.

#### Types of interventions

Experimental interventions theoretically based on guided self-determination or self-determination theory provided face-to-face or digitally by any healthcare professional in any setting. The trials must refer to either guided self-determination or self-determination theory as their primary theoretical framework. Additionally, the trials must use the reflection sheets and the communication forms that are basic to the guided self-determination method.

#### Control group interventions

Control interventions may be ‘no intervention’, wait list or standard care as defined by trialists (e.g. standard healthcare provision). We will also accept attention placebo control [57], which is a control intervention that is not related to enhancing autonomy support but include a similar number of contacts with the interventionists [57].

### Outcomes

#### Primary outcomes

- Quality of life (continuous data) measured by either any validated diabetes-specific questionnaire such as the diabetes quality of life [58] or any validated generic outcome measure such as the WHO-5 questionnaire [59].
- All-cause mortality (dichotomous data).
- Proportion of participants with one or more serious adverse events (dichotomous data), defined as any untoward medical occurrence that resulted in death, was life-threatening, required hospitalisation or prolonging of existing hospitalisation and resulted in persistent or significant disability or jeopardised the patient [60]. If the trialists do not use the ICH-GCP definition, we will include the data if the trialists use the term “serious adverse event.” If the trialists do not use the ICH-GCP definition nor use the term serious adverse event, then we will also include the data, if the event clearly fulfils the ICH-GCP definition for a serious adverse event.

#### Secondary outcomes

- Diabetes distress (continuous data) measured with any validated instruments such as the diabetes distress scale or the problem areas in diabetes scale [61, 62].
- Depressive symptoms (continuous data) measured with any validated instruments such as the Patient Health Questionnaire (PHQ-9) [63] or the hospital anxiety and depression scale [64].
- Proportion of participants with at least one adverse event (dichotomous data) not considered serious [60].

#### Explorative outcomes

- HbA1c (continuous data).
- Motivation measured by the 21-items Treatment Self-Regulation Questionnaire (TSRQ) consists of three subscales measuring the patient’s reasons for taking diabetes medication, checking glucoses, following diet and exercising regularly: (I) autonomous, originating from the self, (II) controlled, pressured or coerced by intrapsychic or interpersonal forces or (III) a-motivated, without intention to change and often feeling unable to change (continuous data).

### Assessment time points

The primary assessment time points for all outcomes will be closest to the end of intervention. We will secondly assess all outcomes at maximum follow-up.

### Search methods for identification of studies

#### Electronic searches

We will search Cochrane Central Register of Controlled Trials (CENTRAL), Medical Literature Analysis and Retrieval System Online (MEDLINE), Excerpta Medical database (EMBASE), Latin American and Caribbean Health Sciences Literature (LILACS), PsycINFO, Science Citation Index Expanded (SCI-EXPANDED), Cumulative Index to Nursing and Allied Health Literature (CINAHL), Social Sciences Citation Index (SSCI), Conference Proceedings Citation Index—Science (CPCI-S), and Conference Proceedings Citation Index—Social Science & Humanities (CPCI-SSH) to identify relevant trials. We will search all databases from their inception to the present. For a detailed example of the search strategy applied in Medline, see Additional file 2. The search strategy for the remaining databases will be given at the review stage.

#### Searching other resources

We will contact the authors of included studies asking for unpublished randomised trials. The reference lists of relevant publications and systematic reviews will be checked for any unidentified randomised trials. Further, we will search for ongoing trials on the following:

- ClinicalTrials.gov (www.clinicaltrials.gov)
- Google Scholar (https://scholar.google.dk/)
- The Turning Research into Practice (TRIP) Database (https://www.tripdatabase.com/)
- European Medicines Agency (EMA) (http://www.ema.europa.eu/ema/)
- US Food and Drug Administration (FDA) (www.fda.gov)
- China Food and Drug Administration (CFDA) (http://eng.cfda.gov.cn/WS03/CL0755/)
- Medicines and Healthcare Products Regulatory Agency (https://www.gov.uk/government/organisations/medicines-and-healthcare-products-regulatoryagency)
- The World Health Organisation (WHO) International
- Clinical Trials Registry Platform (ICTRP) search portal (http://apps.who.int/trialsearch/)
- Cochrane Database of Systematic Reviews
- http://www.evidencebasedpsychotherapies.org/index.php?id=25

Additionally, we will hand search conference abstracts from diabetes conferences for relevant trials and consider relevant-for-the-review unpublished and grey literature trials if we identify these. Reference lists of reviews and meta-analyses retrieved from the searches will also be screened. The latest search will be performed in June 2020 supplemented with ongoing alerts from the databases when new studies within the search matrix are published. We will end inclusion in June 2020.

#### Data collection and analysis

We will conduct the review following The Cochrane Collaboration guidelines [52]. The analyses will be performed by the use of Review manager 5.3 [65]. The analyses will be performed using trial sequential analysis [66] and Stata version 16 [67].

#### Selection of studies

All potentially eligible trials identified in the literature searches will be imported into the systematic review management programme, Covidence [68]. Two authors (ASM) and a co-author will independently screen potentially eligible studies on title and abstract. All full-text studies will be retrieved and independently assessed by the two reviewers. Reasons for exclusion will be reported. Any disagreements will be solved by discussion or by consulting a third author. Trial selection will be displayed in a flow diagram according to the PRISMA-P [56].

#### Data extraction and management

Two authors will independently extract data from included trials. Disagreements will be solved by discussion or by consulting a third author. We will assess duplicate publications and companion papers of a trial together, to evaluate all data simultaneously (to maximise correct data extraction and bias assessment). We will contact all trial authors to specify any missing data, which may not be reported sufficiently or at all in the publication.

#### Trial characteristics

The following data will be extracted: trial design (parallel, factorial, or crossover), number of intervention groups, lengths of follow-up, risk of bias components, and inclusion and exclusion criteria.

#### Participants characteristics and diagnosis

We will extract the following data: number of randomised participants in each intervention group, adherence to intervention, age range (mean or median), sex ratio, type of diabetes, diabetes treatment, number of comorbidities (complications of diabetes/other comorbidities) and socioeconomic status/educational level.

#### Intervention group

We will extract the following data: type of intervention, treatment duration of intervention group, number of sessions (or dose), intensity and treatment format provided to the intervention group.

#### Education and training of the interventionists

The intervention could be provided by any interventionist. Data on who is providing the intervention will be extracted. The training of the interventionists providing the method will be reported. Assessment of fidelity will be reported.

#### Co-intervention characteristics

We will extract the following data: type of co-intervention, treatment duration of co-intervention, number of sessions (or dose) and treatment format.

#### Control group intervention

We will extract the following data: type of control group intervention, treatment duration of control group, number of sessions (or dose), intensity and treatment format provided to the control group. Any reported beneficial and harmful effects of the control intervention will be derived and described.

#### Outcomes

For each outcome, we will extract the number of analysed participants, the number of participants lost to follow-up/withdrawals/crossover in the experimental and the control group.

#### Notes

Funding of the trial and notable conflicts of interest of the trial authors will be reported. Unusual reporting of outcome data will be noted in the ‘Characteristics of included studies’ table. Two reviewers (ASM and co-author) will independently extract and transfer data into Review Manager [65]. Disagreements will be solved through discussion or by consulting a third author.

#### Risk of bias assessment

Risk of bias in included RCTs will be assessed based on the domains described below [52, 69–79]. This assessment will be done separately for each outcome and comparison and will then be considered in relation to overall reliability of the evidence. This will be done in pairs by two independent review authors (ASM and co-author).

#### Random sequence generation

- Low risk of bias: study authors performed sequence generation using computer random number generation or a random numbers table. Drawing lots, tossing a coin, shuffling cards, and throwing dice were adequate if an independent person not otherwise involved in the study performed them.
- Unclear risk of bias: study authors did not specify the method of sequence generation.
- High risk of bias: sequence generation method was not random or quasi-randomised. Such studies will be excluded for the assessment of benefits.

#### Allocation concealment

- Low risk of bias: participant allocations could not have been foreseen in advance of, or during, enrolment. A central and independent randomisation unit controlled the allocation. Investigators were unaware of the allocation sequence (e.g. if the allocation sequence was hidden in sequentially numbered, opaque and sealed envelopes)
- Unclear risk of bias: study authors did not describe the method used to conceal the allocation, so intervention allocations may have been foreseen before, or during, enrolment
- High risk of bias: it is likely that investigators who assigned participants knew the allocation sequence

#### Blinding of participants and personnel

- Low risk of bias: either of the following: no blinding or incomplete blinding, but review authors judged that the outcome was unlikely to have been influenced by lack of blinding *or* blinding of participants and key study personnel ensured, and it was unlikely that the blinding could have been broken
- Unclear risk of bias: either of the following: insufficient information to permit judgement of ‘low risk’ or ‘high risk’; *or* the trial did not address this outcome
- High risk of bias: either of the following: no blinding or incomplete blinding, and the outcome was likely to have been influenced by lack of blinding; *or* blinding of key study participants and personnel attempted, but likely that the blinding could have been broken, and the outcome was likely to have been influenced by lack of blinding

#### Blinding of outcome assessment

- Low risk of bias: either of the following: no blinding of outcome assessment, but review authors judged that the outcome measurement was not likely to be influenced by lack of blinding (we will consider self-reported questionnaires more prone to be affected by lack of blinded outcome assessor and hba1c less likely to be affected by lack of blinded outcome assessor) *or* blinding of outcome assessment ensured, and unlikely that the blinding could have been broken.
- Unclear risk of bias: either of the following: insufficient information to permit judgement of ‘low risk’ or ‘high risk’; or the trial did not address this outcome.
- High risk of bias: either of the following: no blinding of outcome assessment, and the outcome measurement was likely to be influenced by lack of blinding; *or* blinding of outcome assessment, but likely that the blinding could have been broken, and the outcome measurement was likely to be influenced by lack of blinding.

#### Incomplete outcome data

- Low risk of bias: missing data were unlikely to make treatment effects depart from plausible values. The study used adequate methods, such as multiple imputation, to handle missing data or had < 5% missing data.
- Unclear risk of bias: information was insufficient to assess whether missing data in combination with the method used to handle missing data were likely to induce bias on the results.
- High risk of bias: results were likely to be biassed due to missing data.

#### Selective outcome reporting

- Low risk of bias: a protocol is published, or a trial has been registered in a trial register (e.g. clinicaltrials.gov) before or at the time the trial is begun, and the outcome called for in the protocol or trial registration is reported on.
- Unclear risk of bias: study authors did not report all pre-defined outcomes fully, or it was unclear whether study authors recorded data on these outcomes.
- High risk of bias: study authors did not report one or more pre-defined outcomes.

#### Other bias

- Low risk of bias: the trial appeared free of other factors that could have put it at risk of bias
- Unclear risk of bias: the trial may or may not have been free of other factors that could have put it at risk of bias
- High risk of bias: other factors in the trial could have put it at risk of bias

We will judge a trial to be at low overall risk of bias if assessed as having low risk of bias in all of the above domains. We will judge a trial to be at high overall risk of bias if assessed as having unclear or high risk of bias in one or more of the above domains.

We will assess the domains ‘blinding of outcome assessment’, ‘incomplete outcome data’, and ‘selective outcome reporting’ for each outcome result. Thus, we can assess the bias risk for each outcome assessed in addition to each trial. Our primary conclusion will be based on the results of our primary outcome results with overall low risk of bias.

#### Differences between protocol and the review

Any deviations between the published protocol and the review will be reported in the ‘Differences between the protocol and the review’ section of the systematic review.

### Measures of treatment effect

#### Dichotomous outcomes

We will calculate risk ratios (RRs) with 95% confidence interval (CI) for dichotomous outcomes and the trial sequential analysis-adjusted CIs.

#### Continuous outcomes

We will calculate the mean differences (MDs) and consider calculating the standardised mean difference (SMD) with 95% CI for continuous outcomes. We will also calculate trial sequential analysis-adjusted Cis.

#### Dealing with missing data

As specified above, all trial authors will be contacted to obtain any relevant missing data (i.e. for data extraction and for assessment of risk of bias). Secondly, we will investigate the effects of missing data in sensitivity analyses, specified below.

#### Dichotomous outcomes

We will not impute missing values for any outcomes in our primary analysis. In our sensitivity analyses, we will impute data.

#### Continuous outcomes

We will primarily analyse scores assessed at single time points. If only changes from baseline

scores are reported, we will analyse the results together with follow-up scores [52]. If standard deviations (SDs) are not reported, we will calculate the SDs using trial data or Review Manager [65]. We will not use intention-to-treat data if the original paper did not contain such data. We will impute missing values for the continuous outcomes in the sensitivity analyses.

#### Assessment of heterogeneity

To assess any sign of heterogeneity, we will investigate forest plots visually. Secondly, we will assess the presence of statistical heterogeneity by chi^2^ test (threshold *P* < 0.10) and measure the quantities of heterogeneity by the *I*^2^ statistic [80, 81]. Further, we will investigate possible heterogeneity through subgroup analyses and may ultimately decide that a meta-analysis is not warranted [52].

#### Assessment of reporting bias

If ten or more trials are included, we will use a funnel plot to visually assess reporting bias [82]. We are aware of the limitations of a funnel plot (i.e. a funnel plot assesses bias due to the small sample size). From this information, we assess possible reporting bias. For dichotomous outcomes, we will test asymmetry with the Harbord test [83] if *I*^2^ is less than 0.1 and with the Rücker test if *I*^2^ is more than 0.1. For continuous outcomes, we will use the regression asymmetry test [84] and the adjusted rank correlation [85].

#### Unit of analysis issues

We will include randomised clinical trials only. If a trial use a crossover design, only data from the first period will be included [52, 86]. We will include cluster-randomised trials after adjusting the original sample size of the trial to the effective sample size using the intracluster correlation coefficient from the ‘design effect’ [52]. Therefore, there will not be any unit of analyses issues.

### Data synthesis

#### Meta-analysis

We will undertake the meta-analysis according to the recommendations stated in the Cochrane Handbook for Systematic Reviews of Interventions [52], Keus et al. [87] and the eight-step assessment suggested by Jakobsen et al. [88]. Both random-effects meta-analyses [89] and fixed-effect meta-analyses [90] will be used for assessing a potential intervention effect. We will use the more conservative point estimate of the two [88], which is the estimate closest to zero effect. If the two estimates are similar, we will use the estimate with the widest CI. We assess a total of three primary, three secondary outcomes and two explorative outcomes, and we will therefore consider a *P* value of 0.014 or less as the threshold for statistical significance [88]. We will use the eight-step procedure to assess if the thresholds for significance are crossed [88]. As stated, our primary conclusion will be based on results with a low risk of bias [88]. Where multiple trial arms are reported in a trial, we will include the arm(s) relevant for the objective of the review. If two comparisons are combined in the same meta-analysis, we will halve the control group to avoid double-counting [52]. Trials with a factorial design will be included. If a quantitative synthesis is not appropriate due to a small number of trials eligible for inclusion or considerable heterogeneity, the results will be reported narratively.

#### Trial sequential analysis

Traditional meta-analysis runs the risk of random errors due to sparse data and repetitive testing of accumulating data when updating reviews. We wish to control the risks of type I errors and type II errors and thereby the risk of potential false-positive findings of meta-analyses [91]. We will therefore perform trial sequential analysis on the outcomes, in order to calculate the required information size (that is, the number of participants needed in a meta-analysis to detect or reject a certain intervention effect) and the cumulative Z curve’s breach of relevant trial sequential monitoring boundaries [66, 91–98]. More information on trial sequential analysis can be found in the trial sequential analysis manual [98] and at http://www.ctu.dk/tsa/.

For dichotomous outcomes, we will estimate the required information size based on the observed proportion of patients with an outcome in the control group (the cumulative proportion of patients with an event in the control groups relative to all patients in the control groups), a relative risk reduction of 20%, an alpha of 1.4% for all our outcomes, a beta of 10%, and the observed diversity as suggested by the trials in the meta-analysis. For continuous outcomes, we will use the observed SD in the trial sequential analysis, a mean difference of the observed SD/2, an alpha of 1.4% for all outcomes, a beta of 10%, and the observed diversity as suggested by the trials in the meta-analysis.

### Subgroup analysis and integration of heterogeneity

#### Subgroup analysis

The subgroup analyses are moderator analyses that explore effect heterogeneity. Results from subgroup analyses should therefore be interpreted cautiously. The following exploratory subgroup analyses will be conducted when analysing the primary outcomes (Quality of life, mortality and serious adverse events):

##### Participants

1. Type of diabetes. Trials including participants with type 1 compared to trials including participants with type 2.
2. Socioeconomic status defined according to trialists, e.g. educational level. Low socioeconomic status is an umbrella term including educational level and household income which will be used as a proxy for equity if information on educational level is not reported [52]. An equity-focused review must present both relative and absolute differences between groups [15]. We will investigate trials including participants with low socioeconomic status compared to trials including participants with high socioeconomic status.
3. Number of co-morbidities defined as complications of diabetes or other chronic conditions [99].
4. Effect in men compared to women.
5. Effect in adolescents (13 to 18 years) compared to adults >18 years.

##### Intervention

6. Trials investigating self-determination theory compared to trials investigating guided self-determination method.
7. Trials with an experimental intervention above and below the mean difference in intervention length.
8. Trials investigating individual interventions compared to trials investigating group interventions.
9. Type of control intervention (no intervention, standard care or placebo attention control)

##### Risk of bias

10. Trials at overall high risk of bias compared to trials at overall low risk of bias

We will use the formal test for subgroup interactions in Stata [83].

### Sensitivity analysis

#### Dichotomous data

To assess the potential impact of the missing data for dichotomous outcomes, we will perform the two following sensitivity analyses on both the primary and secondary outcomes.

1. ‘Best-worst-case’ scenario: we will assume that all participants lost to follow-up in the experimental group did not die, had no serious adverse events or non-serious adverse events. We will assume the opposite for all participants lost to follow-up in the control group
2. ‘Worst-best-case’ scenario: we will assume that all participants lost to follow-up in the experimental group died, had a serious adverse event or a non-serious adverse event. We will assume the opposite for all participants lost to follow-up in the control group.

We will present the results of both scenarios in our review.

#### Continuous data

When analysing the robustness of a continuous ‘beneficial’ outcome, e.g. quality of life, we will impute the group mean plus two SDs of the group mean. When dealing with a ‘harmful outcome’, we will impute the group mean minus two SDs of the group mean [88]. We will present the results of both scenarios in our review. Other post hoc sensitivity analyses might be warranted if unexpected clinical or statistical heterogeneity is identified during the analysis of the review results [76].

### The quality of evidence

#### Summary of findings table

We will assess the quality of the evidence on the primary outcomes (Quality of life, mortality, serious adverse events), the secondary outcomes (diabetes distress, depressive symptoms and non-serious adverse events) and the explorative outcomes (HbA1c and motivation) using the five GRADE considerations: risk of bias, consistency, imprecision, indirectness and publication bias.

We will assess imprecision using trial sequential analysis or if that is not an option, two authors, ASM and TT, will independently evaluate the quality of the evidence using GRADEpro GDT [100], recommended by the Cochrane Handbook for Systematic Reviews of interventions [52]. Potential disagreements will be solved by an arbiter (co-author). We will report all decisions to downgrade the quality of studies by footnotes to add to the transparency of the decisions. The findings tables will be based on trials with low risk of bias and the results based on all trials.

## Discussion

This is a protocol for a systematic review that aims at synthesising the evidence for the beneficial and harmful effects of guided self-determination or self-determination theory interventions for people with diabetes and comorbidity in any healthcare setting assessed in randomised clinical trials.

The primary outcomes will be quality of life, mortality, serious adverse events, the secondary outcomes diabetes distress, depressive symptoms and non-serious adverse events and the explorative outcomes HbA1c and motivation.

This protocol has several strengths. The predefined methodology is based on the Cochrane Handbook for Systematic Reviews of Interventions [52], the eight-step assessment suggested by Jakobsen et al. [88], trial sequential analysis [66] and GRADE assessment [52, 100]. As such, this protocol considers both risks of random errors and risks of systematic errors.

The limitations of this protocol include the potential for large heterogeneity as a result of including both type 1 and type 2 diabetes and all ways of delivery and interventionists. Therefore, we may ultimately decide that a meta-analysis should be avoided. Moreover, diabetes management always consists of multiple treatment elements [18] and it is likely that different interventions have different effects. Accordingly, if we show a difference between the interventions applying self-determination or self-determination method compared strategies, it will be difficult to conclude what exactly caused the difference in effect. To minimise this limitation, ten subgroup analyses are planned, but results of subgroup analyses should always be interpreted with great caution. The large number of comparisons that increase the risk of type 1 error must also be noted. This issue will be considered when interpreting the results. A further limitation is our exclusion of quasi-randomised studies and observational studies in the assessments of adverse events. By focusing on randomised clinical trials that are unlikely to identify late and rare adverse events, we run the risks of focusing too much on benefits and too little on harms. Therefore, if we identify benefits of the interventions, new systematic reviews focusing on the risks of harms in quasi-randomised studies and observational studies should be conducted to achieve a more balanced evaluation of benefits and harms.

Regarding the health equity subgroup analyses, it has been reported that only 20% report a differential impact of interventions that may increase the social gradient [15]. Thus, we might find that studies do not report on the proxy equity measures for evaluating a differential impact of the interventions described in this protocol [15].

Further, we expect that no trials will have blinded treatment interventionists and participants. Even though blinding of participants should be relatively easy, blinding of treatment providers is theoretically possible but problematic to carry out, especially in psychosocial interventions [57].

## Supplementary Information

**Additional file 1.** PRISMA-P 2015 Checklist.

**Additional file 2.** Search strategy for self-determination theory interventions versus usual care in adults with diabetes (Anne Sophie Mathiesen).

**Additional file 3: Table 1.** The translational potential of the guided self-determination and the self-determination theory.

## Data Availability

We will publish all data including code in the supplementary material of the systematic review.
