# Supplementary material for: Self-determination theory interventions versus usual care in people with diabetes: a protocol for a systematic review with meta-analysis and trial sequential analysis
Source: Syst Rev. 2021 Jan 7;10:12. doi: 10.1186/s13643-020-01566-5 (PMC7791693; doi:10.1186/s13643-020-01566-5)
Supplement: Supplementary file 2 — Additional file 2. Search strategy for self-determination theory interventions versus usual care in adults with diabetes (Anne Sophie Mathiesen). [file 13643_2020_1566_MOESM2_ESM.doc]

# Supplementary file 2: Search strategy for

# Self-determination theory interventions versus usual care in adults with diabetes

# (Anne Sophie Mathiesen)

**MEDLINE Ovid (1946 to April 2020) (hits)**

1. exp Diabetes Mellitus/

2. (diabet* or IDDM or NIDDM or MODY or T1D* or T2D* or insulin* depend* or insulin?depend*).mp. [mp=title, abstract, original title, name of substance word, subject heading word, floating sub-heading word, keyword heading word, organism supplementary concept word, protocol supplementary concept word, rare disease supplementary concept word, unique identifier, synonyms]

3. 1 or 2

4. exp Motivation/px [Psychology]

5. exp personal autonomy/

6. exp Self Care/px [Psychology]

7. exp Empowerment/

8. (self?determination* or empowerment* or life?skill* or consultation near home or reflection?sheet*).mp. [mp=title, abstract, original title, name of substance word, subject heading word, floating sub-heading word, keyword heading word, organism supplementary concept word, protocol supplementary concept word, rare disease supplementary concept word, unique identifier, synonyms]

9. 4 or 5 or 6 or 7 or 8

10. 3 and 9

11. limit 10 to (humans and ("adolescent (13 to 18 years)" or "young adult (19 to 24 years)" or "adult (19 to 44 years)" or "young adult and adult (19-24 and 19-44)" or "middle age (45 to 64 years)" or "middle aged (45 plus years)" or "all aged (65 and over)" or "aged (80 and over)"))

12. (randomized controlled trial or controlled clinical trial).pt. or clinical trials as topic.sh. or trial.ti.

13. (random* or blind* or placebo* or meta-analys*).mp. [mp=title, abstract, original title, name of substance word, subject heading word, floating sub-heading word, keyword heading word, organism supplementary concept word, protocol supplementary concept word, rare disease supplementary concept word, unique identifier, synonyms]

14. 11 and (12 or 13)
