# Supplementary material for: Self-determination theory interventions versus usual care in people with diabetes: a protocol for a systematic review with meta-analysis and trial sequential analysis
Source: Syst Rev. 2021 Jan 7;10:12. doi: 10.1186/s13643-020-01566-5 (PMC7791693; doi:10.1186/s13643-020-01566-5)
Supplement: Supplementary file 3 — Additional file 3: Table 1. The translational potential of the guided self-determination and the self-determination theory. [file 13643_2020_1566_MOESM3_ESM.docx]

Table 1: The translational potential of the guided self-determination and the self-determination theory

|  | **The guided self-determination method** | **The self-determination theory** |
| --- | --- | --- |
| Determining the origin of the theory including development in a clinical setting | Guided self-determination was developed as an empowering decision-making and problem-solving method through a four-stage research program in difficult diabetes care in 1996–2004. | Self-determination theory has been developed from empirical motivational research [31]. It was gradually applied to education, to work organisations, and to health care [36]. |
| The main concepts of the theory | Three grounded theories: 1) keeping life and disease apart [28]; 2) relational potential for change [29]; and 3) a communication and reflection model [27].  General theories: humanistic values theory, self-determination theory, life skills, balanced self-determinism, dynamic judgement building. | Six mini theories:  1 and 2) cognitive evaluation theory I and II; 3) organismic integration theory; 4) causality orientations theory; 5) basic psychological needs theory; 6) goal contents theory [37]. |
| The consistency of the theory including the overarching goal and supportive processes | The supportive processes of guided self-determination method consist of seven stages: 1) establishing a mutual relationship with clear boundaries; 2) self-exploration; 3) self-understanding; 4) shared decision-making; 5) action; 6) feedback from action; and 7) translating evidence for productive patient behaviour in an autonomy-supportive way. | Human beings have three essential psychological needs: autonomy, competence, and relatedness for ongoing psychological growth, integrity, and well-being. The quality of motivation is central to  self-determination theory. The fundamental distinction is between autonomous and controlled forms of motivation. The predominant feeling is ‘willingness’. In controlled motivation, the predominant feeling is pressure, which is often associated with ambivalence or resistance. |
| The degree of generalisability and parsimony of the theory including general principles, strategies or tools for engaging peoples | The person with diabetes-health care provider relationships can be released through self-reflection, mutual reflection, shared decision-making, dynamic judgment building, and autonomous motivation leading to self-concordance. Requires changes by both health care professional and person with diabetes | 1) Autonomy support: *relevance*,  *respect, choice, and avoidance of control.*  2) Structure: *clarity of expectations*, o*ptimal challenge, feedback, instrumental and practical skills-training*, *guidance and support*.  3) Involvement (support for relatedness):  *empathy, affection, attunement*, *dedication of resources, dependability.* |
| How the theory is tested in empirical research including reported effects | The guided self-determination method has not yet been systematically reviewed or meta-analysed. The method has been tested in 4 randomised clinical trials and people with type 1 diabetes. Results showed significant improvement in glycemic control and life-skill, reduced diabetes distress, and improved diabetes competences [30, 38]. | Three meta-analyses [18, 19, 39] including diverse participants, primarily health people. Self-determination theory-based interventions have been tested in people with type 2 diabetes, showing an effect in women on HbA1c and quality of life [40]. A study reports effect on eating behavior but not on HbA1c in adolescents with type 1 diabetes [41]. |
| The usefulness and practicability of the theory including health care professionals’ background and training and fidelity assessment | The health care professionals undergo 32 hours of structured and supervised training to become certified guided self-determination facilitators. They document their ability to use the reflection sheets and communication skills in two full courses with patients. Figures from grounded theories are used as fidelity assessment tools. | No formal training of personal or fidelity testing.  Fidelity tools from motivational interviewing have been applied [19]. |
